# Fistulous Openings in the Perineum

**Published:** 1849-03

**Authors:** Bransby B. Cooper

**Affiliations:** Surgeon, and Lecturer on Surgery at Guy’s Hospital


### SURGERY.

*Fistulous openings in the Perineum.* By Bransby B. Cooper, F.R.S. Surgeon, and Lecturer on Surgery at Guy's Hospital.—Retention of urine, whether arising from permanent stricture, the presence of calculus, or, indeed, from any cause which prevents the flow of the urine, may be spontaneously relieved by ulceration of the urethra behind the cause of obstruction, from which extravasation of urine, and subsequently abscesses, must necessarily result, and fistulous openings being established, the patient is relieved by the flow of urine through them. Such a condition must not, however, be allowed to remain; and it is quite clear that the fistula cannot be cured without the stricture being divided so as to restore the natural canal for the passage of the urine. Puncture of the bladder in such cases would, in my opinion, be quite ineffective in relieving the patient, and the cure can only be produced by dividing the stricture, in perineo, as I have already described, and by freely laying open all the sinuses, however numerous they may be. A gentleman, from Barbadoes, came under my care, who had not only been the subject of stricture for several years, but had also fistulous openings into the perineum, scrotum, and even into the rectum. In this case I first divided the stricture in perineo, and passed a large catheter snto the bladder; I then laid open all the sinuses, and divided the sphincter ani muscle: in three months the patient was perfectly cured.

*Abscesses in the perineum* sometimes result from stricture, even when the obstruction is not sufficient to produce actual retention of urine. These abscesses occur from the dilatation of the urethra behind the stricture, leading to ulceration of its structures, and consequent infiltration of urine: in such cases swelling in the perineum soon results, and rigours supervene, indicating the formation of pus: a free opening into the perineum should be immediately made, and the catheter passed, if practicable, along the natural passage of the urethra into the bladder: should you not be able to effect this at the time, repeated gentle efforts must be made to restore the normal continuity of the canal. Such ulceration of the urethra may, however, occur, as I have already said, without being produced by an impermeable stricture, so that it frequently happens, in such cases, that the catheter passes readily into the bladder without meeting any insuperable obstruction; but this should never be attempted until the abscess be opened, as the accumulated matter itself might cause considerable impediment to the passage of the instrument. The following case affords an example of this fact:—I was sent for to see a patient who was suffering from retention of urine, of which the symptoms were so urgent that I immediately attempted to pass a catheter; not succeeding, however, in relieving the patient, I proceeded to examine the perineum, where I discovered a tumour of considerable size: in this I made an incision, and a quantity of pus and urine were immediately evacuated. As the patient stated that he had been the subject of stricture for many years, I considered it better to open at once the membranous part of the urethra; I therefore passed a female catheter into the bladder, and, drawing off the urine, relieved the patient from the symptoms arising from the retention; I next passed a male catheter along the natural passage of the urethra, as a preliminary to the division of the stricture; to my great surprise, the instrument readily passed on, and when the female catheter was withdrawn, at once entered the bladder: this circumstance showed that if I had attempted to pass the male catheter before I divided the membranous portion of the urethra, I should have found the more formidable part of the operation to be altogether unnecessary. The experience I derived from this case has since often prevented me from cutting into the membranous part of the urethra, after opening an abscess in perineo, without first attempting to pass the male catheter along the natural course of the urethra: such a precaution is, indeed, rendered doubly necessary by the fact that abscesses in the perineum may result from external injury, without any other im plication of the urethra on the canal than that arising from the mere pressure of accumulated matter, the evacuation of which immediately relieves the symptoms.

*Diseases of the prostate gland.*—It is somewhat difficult to decide whether the prostate gland is most important as a urinary or as a generative organ; as its diseases interfere, however, with the performance of both these functions, it may rationally be viewed in relation to either of them. The prostate gland is composed of numerous follicles connected by a dense cellular membrane; but it is not furnished with a distinct capsule, which circumstance probably accounts, at least in some measure, for its tendency to undergo sudden enlargement. The excretory ducts of the prostate gland, which are from fifteen to twenty in number, terminate in the prostatic portion of the urethra, by the sides of the verumontanum; and if the urethra be laid open at this part, the secretions may be seen to exude when the gland is subjected to pressure. The natural form of the prostate gland is that of a chestnut, divided by a raphe or longitudinal fissure into two lateral lobes. The fissure is much more defined on the inferior than on the superior surface; at the posterior and inferior part of the gland there exists a small process, which connects the lateral lobes, and which is sometimes termed the third lobe of the prostate. The prostate gland is remarkable for its liability to undergo enlargement at advanced periods of life; and this complaint is, indeed, very common and scarcely to be looked upon in the light of a disease. The effects of enlargement of the prostate are, however, very important, as it produces an impediment to the passage of the urine, and is by far the most frequent cause of retention of urine in old age. The urethra traverses the prostate gland, but not exactly through its centre, being nearer to the upper than to the under surface; this is termed the prostatic portion of the urethra, and in it the ducts of the vesiculæ seminales and vasa deferentia have their terminations. The urethra, where it passes through the prostate, is subject to a disease which cannot, however, be regarded as an affection of the gland itself; the symptoms of the complaint are—pain at the neck of the bladder, aggravated during micturition, and a degree of difficulty in passing the urine, which sometimes leads to a suspicion of the presence of stricture. If the supposed obstruction be sought for by the bougie, intense pain is experienced as the instrument enters the prostate, and the patient complains of a great aggravation of the symptoms "in coitu:" the latter peculiarity forms the principle diagnostic mark of the disease, which consists in a morbid sensibility of the extremity of the vasa deferentia where they enter the urethra. This affection is produced by enlargement of the extremities of the vasa deferentia, which throw out papillæ-like projections that are extremely sensitive, and form obstructions to the passage of the urine and serum. The caustic bougie is the best remedy for this complaint, to which I believe Lallemand was the first to direct the attention of surgeons. I have two or three times seen it, and recognized it from its seat being in the prostatic portion of the urethra (a part very seldom the subject of stricture,) and from the pain in coition, and during the passage of the urine, as well as from the sympathetic tenderness of the testicle itself. The application of caustic proved sufficient to effect a cure in all these cases. The prostate gland is liable to inflammation, which is very often induced by the extension of gonorrhœal inflammation; it is indicated by a sense of pain in the perineum, extending into the region of the rectum, the pain being greatly aggravated while the patient is in the sitting posture. The bladder becomes effected, if immediate relief be not afforded to the inflamed gland, and even retention of urine often supervenes. The treatment consists in the application of leeches to the perineum, cupping in the loins, keeping the patient in the recumbent posture, suppositories, the use of diluents, and the removal of accumulated fæces from the large intestines. If such means be employed, the attack generally yields at once; but if they be not sufficiently active, the inflammation may acquire a chronic form, which it will be found extremely difficult to overcome, and which, indeed, frequently leads to abscess and very protracted suffering.

*Abscess in the prostate gland.*—When an abscess has formed in the prostate, the pain becomes completely changed in its character, being then rather an obtuse throbbing sensation, which is much aggravated during the act of defecation; rigors also supervene, and sometimes the shivering fit is so distinctly intermittent, that the affection is liable to be mistaken for ague. The difficulty in passing the urine is also increased by the formation of matter, and an examination of the prostate gland should now be made per rectum; when it is said fluctuation may sometimes be discovered: I must, however, acknowledge that I could never detect it by this method of investigation. In abscess of the prostate, the catheter must be employed, notwithstanding the pain which its introduction produces, and although an obstruction is felt in passing the instrument through the prostate: the resistance is not similar to that of stricture; moreover, as stricture never occurs at this part, the diagnosis of prostatic abscess becomes comparatively easy. There is another point worthy observation; in abscess, the obstruction returns immediately after the removal of the catheter or bougie, to the same extent as before its introduction: this is not the case with stricture of the urethra, Should the duration of the symptoms be greatly protracted, and the constitution of the patient deteriorated by continued suffering, the abscess should be opened by making a deep incision into the perineum, and then passing a bistoury into the gland to evacuate the matter. In this operation, the urethra should be carefully avoided, so that the matter may pass off by the factitious opening alone. Abscesses in the prostate sometimes burst spontaneously, either into the urethra or rectum, and this is generally indicated, in the case of the former, by the urine being mixed with pus, and by the immediate relief from pain: but when the tumor bursts into the rectum the only probable sign of the evacuation of the matter would be the complete cessation of all suffering. The opening between the urethra and prostrate, formed by the bursting of an abscess, is frequently very difficult to cure, owing to the urine filtering in the prostate, and keeping up a constant irritation: this difficulty may, however, be overcome by the gentle introduction of a catheter, to draw off the water for a few days,—until, indeed, the abscess has granulated. Should there be much difficulty in healing an opening between the rectum and the prostate, owing to the intrusion of the feculent matter into the gland, the division of the sphincter ani seems to be the best means of affecting the cure of the fistula. Sir Benjamin Brodie has recorded some cases of abscess in the prostate gland in children, which occurred in his own practice. I have never met with a case myself, and consider it rather a disease of the adult, than one either of youth or old age.

*Ulceration of the prostate gland.*—This sometimes results from the inflammation, but much more frequently, I believe, from the presence of calculi in the prostate: it is attended by the most excruciating pain, and by occasional bleeding. Caustic is the best remedy for this disease, and may be passed down by a catheter converted into a "port caustique;" in two or three instances in which I have known it to be used, it has been productive of the most beneficial effect.

*Calculi in the prostate.*—This disease is not very uncommon, but frequently remains unsuspected until a small calculus ulcerates into the urethra, causing retention of urine: sometimes, however, premonitory symptoms present themselves, resembling those already described as attendant upon inflammation of the gland. Some time ago I was sent for to visit a gentleman in Westbourne Terrace, who had been suddenly seized with retention of urine; he told me that he suspected the presence of a stone, as a few days before he had passed a small one during micturition. On introducing a catheter into the urethra. I met with an obstruction, just anterior to the bulb, and I did not attempt to overcome it, on account of the danger of pushing the calculus back into the bladder if it should prove to be one. The symptoms of retention not being very urgent, I ordered a hot bath and a large dose of opium, and four hours after he passed a calculus weighing five grains: it proved, upon analysis, to consist chiefly of phosphate of lime. The formation of stone in the prostate is not, however, always attended by so little inconvenience; the most acute suffering I ever witnessed, and which indeed led ultimately to the death of the patient, was in a case of this kind. The patient was under the care of Sir Astley Cooper, who attempted to remove the calculi from the prostate by means of the scoop, having cut down upon the gland, as in the operation for stone; but as the calculi were contained within the follicles of the gland, so as to be somewhat sacculated, it was impossible to remove them all, and the suffering of the patient remained unrelieved; indeed, his constitution and mind both at length became so worn by the continual agony, that he put an end to his existence.

*Enlargement of the prostate in old age.*—This can scarcely be regarded as a disease, but appears to be the result of a change inseparable from old age. It certainly sometimes attacks individuals at comparatively early life, but such persons always manifest unequivocal signs of premature decrepitude. The enlargement seems to be true hypertrophy, as it is rarely attended by any alteration of texture, although I have in some few cases found the gland softer, and in others harder, than natural. The symptoms in enlargement of the prostate gland depend with respect to their urgency upon the size it has acquired; they are, sense of weight in the perineum, intolerance of pressure from the hardness of a seat; difficulty in passing the urine, and also in voiding the fæces, which will be found flattened by the encroachment of the hypertrophied gland on the rectum. At this stage of the complaint, retention of urine occasionally supervenes, rendering the introduction of a catheter necessary. This operation should be performed with the utmost gentleness, as the slightest flow of blood would cause decomposition of the urine, and consequent aggravation of all the symptoms. An elastic gum catheter should always be used for drawing off the water, and, if possible, it should be introduced without a stilette; leeches should be applied to the perineum; the rectum emptied by means of enemata; and suppositories, recumbent position, and soothing remedies, employed. I have also found colchicum of great use in such cases, and believe that its beneficial influence arises from the circumstance that this disease frequently attacks subjects of a gouty diathesis. I usually prescribe the colchicum in the following form:—R. Ext. Colchici Acet. gr. j.; Pil. Hydrarg. gr. j.; Pulv. Doveri. gr. v.; Ext. Colocynth. Co. gr. iij. M. Ft. pil. bis quotidie sumenda.

As the complaint takes its origin from a particular epoch of life, nothing more than relief of the symptoms can be expected; but nevertheless, by a judicious system of diet, by keeping the patient from excess of bodily exertion, and from vicissitudes of temperature, his life, which was scarcely supportable under the violent symptoms of the disease, is rendered comparatively free from pain and inconvenience.

It does not always happen that the whole of the prostate gland becomes hypertrophied in old age: but very frequently the third lobe only is affected, or perhaps it may more properly be said that a new development arises; for in a state of health, at the adult period, the third lobe is scarcely perceptible. When this third lobe enlarges, it presses the inferior region of the bladder or "trigone" upwards above the commencement of the urethra in the bladder, preventing the evacuation of the urine, and consequently producing retention. Nor is this the only inconvenience; for by the raising of the bladder immediately behind the prostate, a kind of reservoir is established below the entrance of the urethra; and, in the effort to empty the bladder, a portion of its contents is always left: this becomes specifically heavier than the newly-secreted urine, which does not intermix with it; and, after a time, the retained urine undergoes decomposition, which gives rise to very urgent symptoms—such as frequent desire to make water, tenesmus, deep-seated pain in the perineum, and liability to positive retention. It is quite clear that these symptoms cannot be removed while the exciting cause remains; the fœtid urine must therefore be immediately drawn off by means of the catheter. In such cases there is, however, a difficulty in passing the instrument, as the enlarged lobe offers some degree of obstruction to its passage, and this is is only to be overcome by employing a longer and larger catheter than that usually made use of: this instrument is generally termed the prostatic catheter. The mode of introducing the catheter in such cases is similar to that in ordinary practice, until it arrives at the point of obstruction, when the penis and instrument are both to be drawn forwards for the purpose of straightening the urethra; the handle of the catheter is then to be considerably depressed, so as to tilt up the point, and it is then pressed onwards into the bladder. But, having effected this, the urine would only be drawn off to the level of the urethra, and the heavier fluid would still remain, unless further means were employed for its removal. The cleansing of the bladder may be effected by injecting it with tepid water, by means of a syringe; and an improved instrument has been invented for this purpose, by which a continuous current is kept, the same stroke of the piston removing one quantity, and supplying a fresh one. Constitutional remedies must not be neglected; and, when an alkaline state of the urine exists, medicines of an acid character are generally indicated. Among the most efficacious of these will be found the following:—℞ Nitro-Hydrochlor. Acid. gtt. iij.; Syr-Papav. ʒiij.; Inf. Colomb. ℥iss. M. Ft. haustus ter quotidie sumendus. In addition to this, an opiate suppository at bed time will often be found of great advantage; but if an acid condition of the urine be not thus restored, Liq. Potassæ will frequently be found capable of re-establishing the normal acid state: this anomaly has been accounted for by Dr. G. O. Rees, on the supposition that the alkali renders the secreted urine less irritating to the mucos membrane of the bladder, and preventing the secretion of alkaline mucus, from which the urine had acquired its abundant preponderance of alkali.—*London Medical Gazette.*

